# Displacement Rate Effects on the Mode II Shear Delamination Behavior of Carbon Fiber/Epoxy Composites

**DOI:** 10.3390/polym13111881

**Published:** 2021-06-06

**Authors:** Kean Ong Low, Mahzan Johar, Haris Ahmad Israr, Khong Wui Gan, Seyed Saeid Rahimian Koloor, Michal Petrů, King Jye Wong

**Affiliations:** 1 School of Mechanical Engineering, Faculty of Engineering, Universiti Teknologi Malaysia, Skudai 81310, Johor, Malaysia; kolow@mmu.edu.my (K.O.L.); haris@mail.fkm.utm.my (H.A.I.); 2 Centre for Advanced Materials and Green Technology, Faculty of Engineering and Technology, Multimedia University, Jalan Ayer Keroh Lama, Bukit Beruang 75450, Melaka, Malaysia; 3 Faculty of Engineering and Science, Curtin University Malaysia, Miri 98009, Sarawak, Malaysia; mahzan.johar@curtin.edu.my; 4 School of Engineering, University of Southampton Malaysia, Kota Ilmu Educity @ Iskandar, Iskandar Puteri 79200, Johor, Malaysia; K.W.Gan@soton.ac.uk; 5 School of Engineering, University of Southampton, Highfield, Southampton SO17 1BJ, UK; 6 Institute for Nanomaterials, Advanced Technologies and Innovation (CXI), Technical University of Liberec (TUL), Studentska 2, 461 17 Liberec, Czech Republic; 7 Department of Aerospace Engineering, Faculty of Engineering, Universiti Putra Malaysia, Serdang 43400, Selangor, Malaysia; 8 Technical University of Liberec (TUL), Studentska 2, 461 17 Liberec, Czech Republic; michal.petru@tul.cz

**Keywords:** carbon/epoxy composite, Mode II delamination, cohesive zone model, displacement rate, fractography

## Abstract

This paper studies the influence of displacement rate on mode II delamination of unidirectional carbon/epoxy composites. End-notched flexure test is performed at displacement rates of 1, 10, 100 and 500 mm/min. Experimental results reveal that the mode II fracture toughness *G_IIC_* increases with the displacement, with a maximum increment of 45% at 100 mm/min. In addition, scanning electron micrographs depict that fiber/matrix interface debonding is the major damage mechanism at 1 mm/min. At higher speeds, significant matrix-dominated shear cusps are observed contributing to higher *G_IIC_*. Besides, it is demonstrated that the proposed rate-dependent model is able to fit the experimental data from the current study and the open literature generally well. The mode II fracture toughness measured from the experiment or deduced from the proposed model can be used in the cohesive element model to predict failure. Good agreement is found between the experimental and numerical results, with a maximum difference of 10%. The numerical analyses indicate crack jump occurs suddenly after the peak load is attained, which leads to the unstable crack propagation seen in the experiment.

## 1. Introduction

Delamination is one of the major failure mechanisms in composite laminates due to their low interlaminar strength [1,2,3,4]. For composite materials used in aeronautical applications, barely visible impact damage (BVID) could be generated under low energy impact loading [5]. BVID could happen at lamina level as well [6]. During the impact loading, the mode II shearing mode is particularly important as large through-thickness shear stresses are induced due to bending of the composite structures [1]. In addition, the material response and properties of polymeric materials are commonly known to be rate-dependent [7,8]. Hence, it is essential to understand the influence of loading speed on mode II interlaminar fracture toughness.

In the literature, it has been reported that for zero dominated [(0/−45/0/45)_3S_]_S_ IM7/8552 carbon/epoxy composites, mode II interlaminar fracture toughness *G_IIC_* was increased up to 46% (from an average value of 663 N/m to 970 N/m) when tested in the range of 0.5–3.3 × 10^5^ mm/min [9]. At the testing speed of 6 × 10^3^ mm/min, the *G_IIC_* value of unidirectional carbon reinforced composites tested was found to increase by 20% compared to the one tested at 3 mm/min [10]. In the study by Cantwell [8], within a smaller range of 0.1–500 mm/min, an increment of nearly 20% and 45% was obtained for unidirectional carbon/brittle epoxy and woven carbon/toughened epoxy composites, respectively [11].

Some studies on the other hand showed decreasing of mode II fracture toughness in carbon/epoxy composites with loading speed. An average of 20% decrement has been reported for unidirectional T300/2500 and IM600/133 carbon/epoxy composites within 0.2–1.2 × 10^6^ mm/min [12]. Also, a decrement of approximately 10% was found in unidirectional Texipreg HS 160 RM carbon/epoxy composites when tested at 2 and 100 mm/min [13]. It is worth to note that the percentage of drop was similar for specimens tested at all temperatures of −30, 20 and 80 °C.

In unidirectional GV 170 U carbon reinforced SR 8100/SD 8822 epoxy composites, *G_IIC_* was found to be invariant when tested within 5–1.14 × 10^4^ mm/min [14]. The influence of displacement rate (1–3 × 10^5^ mm/min) on *G_IIC_* was also found to be insignificant for unidirectional T-400 carbon/epoxy composites [15]. Similar observation was reported for untufted carbon/epoxy non-crimp fabric composites within the speed of 1–4.2 × 10^5^ mm/min [16].

From the above literature review, it has been found that the influence of displacement rate on mode II interlaminar fracture toughness is mixed. It is highly dependent on the type of epoxy and the loading conditions, and mechanical testing is often needed before a semi-empirical model or equation can be proposed to describe the overall behavior for a particular material system. It is also important to find a general model that is able to widely describe the mixed or diverse relationship between *G_IIC_* and displacement rate found in the literature.

In this paper, the displacement rate effects on mode II delamination behavior of an out-of-autoclave unidirectional carbon/epoxy composite is characterized. The displacement rate effect of the mode II delamination is investigated using end-notched flexure (ENF) test conducted at four displacement rates, i.e. 1, 10, 100 and 500 mm/min. Mode II fracture toughness *G_IIC_* is calculated using the compliance calibration (CC) method following ASTM D7905/D7905M-14 [17]. The delaminated surfaces are analyzed using scanning electron microscope to look into the fracture mechanisms at different displacement rates. Subsequently, a rate dependent model for *G_IIC_* is proposed, where the results from the current study as well as from the open literature are fitted to demonstrate its general applicability for composite materials. Finally, validation of the simulation process [18,19] and numerical analysis using finite element models are performed through comparison of the global force-displacement curves and crack growth behavior at the delamination interface [4,20,21].

## 2. Materials and Methods

### 2.1. Materials and Specimens

**Figure 1:**
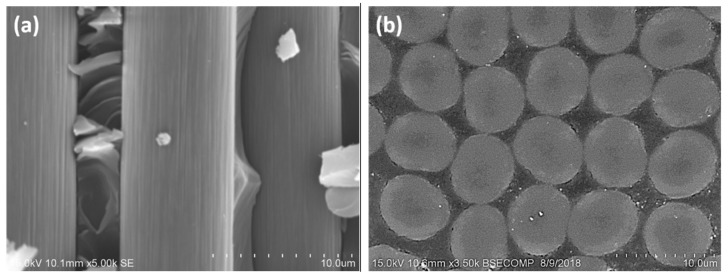
(**a**) Longitudinal view and (**b**) cross-sectional area view of the carbon fiber.

The unidirectional carbon/epoxy prepreg used in this study has a nominal ply thickness of 0.15 mm. The average fiber volume fraction is 65.7 ± 6.3%, while the carbon fibers are continuous in length and have an approximately circular cross-sectional area, with an average diameter of 6.8 μm (Figure 1). All specimens are prepared and supplied by X Plas Singapore. A unidirectional composite plate with [0]_20_ is fabricated using hand lay-up technique. To generate the pre-crack, a 15 μm thick Teflon film is placed at the mid-plane of the plate. The composite laminate is then hot-pressed, giving a final average plate thickness of 3 mm. It is then cut into specimens of 20 mm wide using a computer numerical control (CNC) cutter. Table 1 lists the lamina properties for the carbon/epoxy composite that have been reported in a previous study [22].

### 2.2. Mode II Interlaminar Fracture Toughness Test

**Figure 2:**
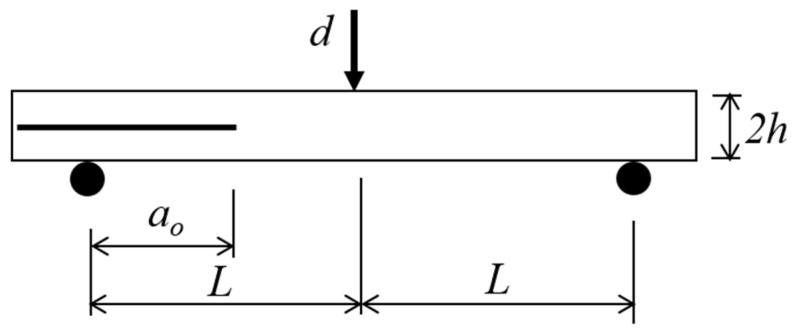
Schematic diagram of ENF test setup.

Mode II shear loading test is conducted using end-notched flexure (ENF) test according to ASTM D7905/D7905M-14 [17]. Figure 2 illustrates the test setup configuration. The total thickness of the specimen *2h* is 3 mm, the initial crack length *a_o_* is 30 mm and the half span length *L* is 50 mm. This gives the initial crack length to half span length ratio as 0.6. Interlaminar fracture toughness tests are carried out at displacement rates of 1, 10, 100 and 500 mm/min until delamination occurred. 500 mm/min is the highest loading speed offered by the Instron Universal Testing Machine 5982 and is estimated to be the same range of speed in the event of tool mishandling or tool drop during maintenance and assembly of aircraft structures [5]. All tests were displacement controlled with the load cell capacity of 5 kN. Three specimens were tested for each crosshead speed. All tests were performed at the ambient conditions.

### 2.3. Morphology Study

After the specimens were tested, one of the specimens from each displacement rate was inspected under the scanning electron microscope (Philips XL40, FEI, Hillsboro, ON, United States) to investigate the damage evolution and morphology of the delaminated surface. The delaminated surfaces are gold coated by 134 Bio-Rad Polaron Division before SEM images are taken. 

### 2.4. Data Reduction Method

The calculation of the mode II fracture toughness *G_IIC_* is based on the Irwin–Kies [23] equation:

(1) $$G_{IIC}=\frac{P_{C}^{2}}{2B}\left(\frac{dC}{da}\right)$$

where *P_C_* is the critical load, *B* is the width of the specimen, *C* is the compliance (inverse of the initial linear slope of the load-displacement plot) and *a* is the crack length. The data reduction scheme follows ASTM D7905/D7905M-14 [17], where the compliance calibration model is written as:

(2) $$C=C_{2}a^{3}+C_{1}$$

In Equation (2), *C_2_* and *C_1_* are obtained through fitting of *C* – *a^3^* plot. Substituting the derivative of Equation (2) into Equation (1) yields:

(3) $$G_{IIC}=\frac{P_{C}^{2}}{2B}\cdot 3C_{2}a^{2}$$

The initial crack length is fixed at 30 mm for all displacement rates. In order to generate the compliance plot, additional specimens are tested at crack lengths of 20, 25, 35 and 40 mm within the linear load-displacement region, at 1 mm/min displacement rate only. It is because the compliance of the specimens is found to be independent of the displacement rates between 1 mm/min–500 mm/min, which will be further described in Section 3.1. The same observation is also found in mode I delamination of the same carbon/epoxy composite [22]. Therefore, the same data reduction scheme for quasi-static loading as described in ASTM D7905/D7905M-14 [17] is used for higher speeds.

## 3. Experimental Results and Discussion

### 3.1. Force-Displacement Curves

**Figure 3:**
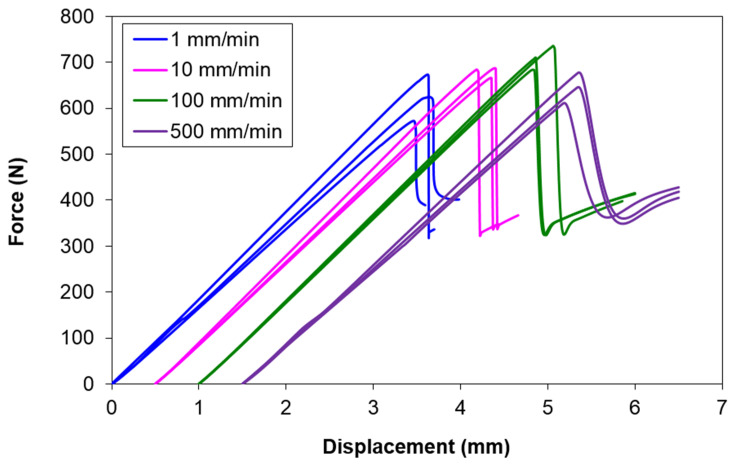
Force-displacement curves of ENF specimens at all crosshead speeds. The curves at each speed are offset for clarity.

Figure 3 shows the force-displacement curves of the ENF specimens at crosshead speeds of 1, 10, 100 and 500 mm/min. During the early stage of loading, the force increases linearly with the imposed displacement. The load drops abruptly after the peak load, indicating unstable delamination crack growth. At all displacement rates, the slopes at the initial loading region are close to each other. The average slopes (or stiffness) are 180, 183, 185 and 175 N/mm for 1, 10, 100 and 500 mm/min, respectively. All slope values are measured within the applied displacement range of 1–3 mm. By using the average slope at displacement rate 1 mm/min as the reference, the maximum difference between them is only 3%. This implies that the stiffness of this composite is not sensitive to displacement rate from 1 mm/min to 500 mm/min. This is similar to the observation by Cantwell [11], which was also highlighted by Yasaee et al. [9].

### 3.2. Mode II Interlaminar Fracture Toughness

**Figure 4:**
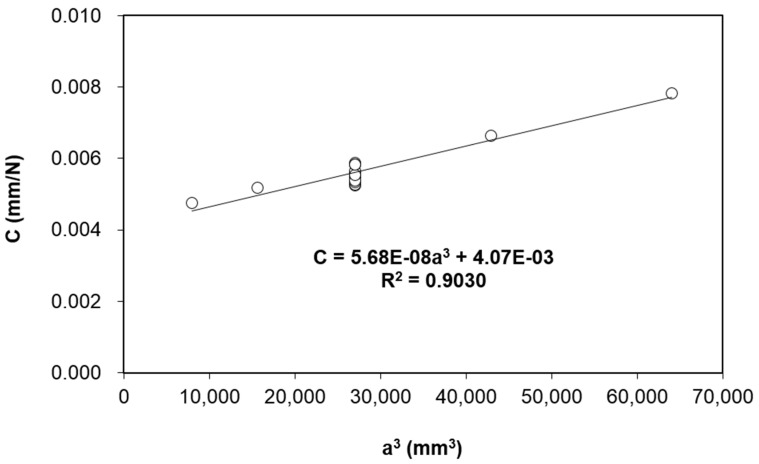
Compliance plot of the ENF test at different crack lengths.

Figure 4 depicts the compliance plot of the ENF tests. The best fit parameters are *C_2_* = 5.68 × 10^−8^ mm^−2^∙N^−1^ and *C_1_* = 4.07 × 10^−3^ mm∙N^-1^, with *R*^2^ = 0.9030. The same *C_2_* value will be used to compute *G_IIC_* for all displacement rates, as the compliance is similar at all displacement rates as discussed in Section 3.1. The average *G_IIC_* values given by Equation (3) are listed in Table 2. With respect to 1 mm/min displacement rate, the differences of *G_IIC_* are 33%, 45% and 20% for 10 mm/min, 100 mm/min and 500 mm/min, respectively. Their differences are statistically significant based on their standard deviations with respect to the quasi-static displacement rate (1 mm/min), but the differences among the higher displacement rate cases are less significant. It is also worth to note that the *G_IIC_* values of the composite studied in this work are comparable to some of the composites reported in the literature [9,11,15,16,24].

**Figure 5:**
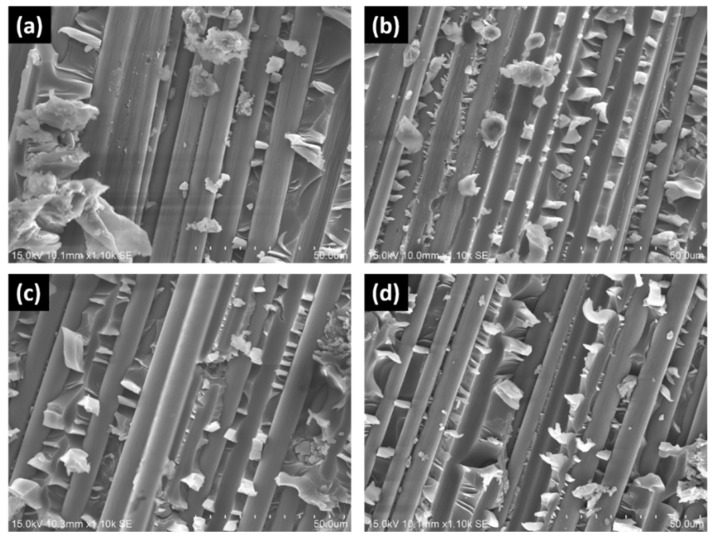
Scanning electron micrographs of the ENF specimens at (**a**) 1, (**b**) 10, (**c**) 100, and (**d**) 500 mm/min.

The SEM images in Figure 5a illustrates that fiber-matrix debonding is the dominant failure mechanism at crosshead displacement speed of 1 mm/min, where clean and smooth fiber surfaces are observed. This indicates the adhesive failure of the fiber/matrix interface, which was also mentioned by some other researchers [25,26]. In addition, some matrix cracking is noticeable as well. On the other hand, it is obvious that the delaminated surfaces at higher displacement rates (Figure 5b–d) are rougher with more matrix hackles, which are not as abundant in the case of 1 mm/min. These are shear cusps due to matrix deformation and they are commonly observed in brittle matrix composites under mode II loading [9,26,27,28,29,30,31]. Adhesive failure at low speed and shear cusps formation at higher speed were also reported for E-glass/epoxy and E-glass/vinyl ester composites [25]. More energy dissipation due to the higher number of shear cusp formation in the viscoelastic matrix is postulated to be the reason for the 20–45% of increment in *G_IIC_* at high loading rates. The SEMs support the notion that the energy release in mode II delamination is matrix dominated [25,30,31], and fiber/matrix debonding has negligible influence on the mode II interlaminar fracture toughness [25]. The similarity in the SEM images of the post-failure fracture surfaces among the cases of higher displacement rates (Figure 5b–d) explains the slight difference of *G_IIC_* values at these higher displacement rates. Similar to the observation by other researchers [28,29,30]. Plastic deformation in the matrix is not obvious as the epoxy used is a highly cross-linked brittle polymer [29].

**Figure 6:**
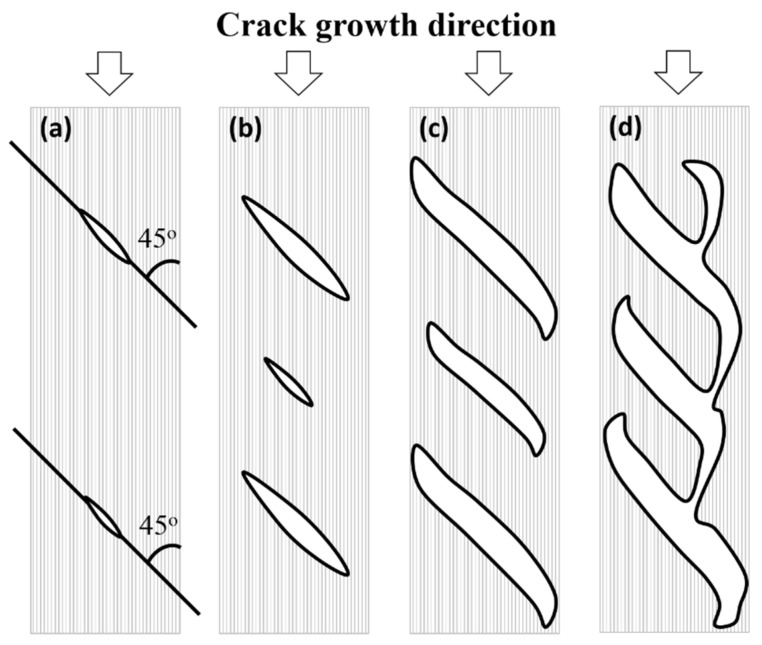
Schematic diagrams of crack formation in ENF specimens (**a**) micro-cracks (**b**) extension of micro-cracks and formation of a new crack (**c**) crack growth near shear band limit (**d**) coalescence of cracks [32].

Figure 6 describes the crack formation process under shear mode delamination that is commonly described by other researchers [32]. Due to the stress concentration at the crack tip, multiple micro-cracks will be initiated from there, which is also the resin-rich region [33]. Figure 6a illustrates that the micro-cracks are mainly formed at 45^°^ with respect to the crack growth direction because that is the orientation of maximum tensile stress. Subsequent increment in the shear loading leads to the growth of the micro-cracks and formation of new cracks (Figure 6b until the cracks reach the limit of the shear band (Figure 6c). Finally, coalescence of the multiple cracks (Figure 6d) leads to the crack growth along the interface plane. Therefore, matrix failure by shear is the major fracture mechanism that leads to the separation of the two neighboring plies. Not only that, during the crack propagation process, the matrix cracking could also be extended along the interface between the fibers. When this happened, the neighboring fibers were separated, which is known as fiber/matrix interface debonding.

**Figure 7:**
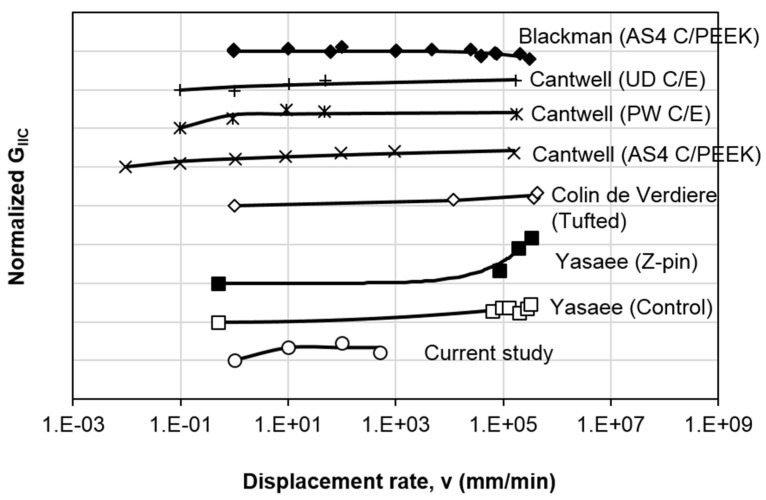
Comparison of the variation of normalized mode II fracture toughness with respect to displacement rate.

The variation of the normalized mode II fracture toughness with respect to the normalized displacement rate of this study is plotted in Figure 7. They are normalized with respect to the values at the reference quasi-static values (1 mm/min). The data can be well-fitted using the proposed equation:

(4) $$\frac{G_{IIC}(v)}{G_{IIC,QS}}=1+m{\left[\ln\left(\frac{v}{v_{QS}}\right)\right]}^{\zeta }$$

In Equation (4), *G_IIC_ (v)* is the mode II interlaminar fracture toughness at loading speed *v*, the subscript *QS* refers to the *G_IIC_* at reference quasi-static loading (or the lowest loading speed), and *m* and *ζ* are the fitting parameters. This equation is a modified version from the one proposed by May [7], which is also used by Machado et al. [13,24]. The original equation does not have the exponent *ζ*, in which case it considers that the normalized fracture toughness always varies linearly with *ln(v/v_QS_)*, which might not be true for all types of epoxy. It can be demonstrated that the original equation does not capture satisfactorily the results from the current study and some in the open literature. Hence, an exponential parameter *ζ* is added to account for the nonlinear variation. Some of the test data from the literature is also shown in Figure 7 and fitted with Equation (4) to demonstrate its general applicability. Note that only the test data having a minimum of four data points from the literature discussed in Section 1 are included in Figure 7. It is because the test results with only two data points does not give a nonlinear behavior, and it is meaningless to fit the test results with three data points where Equation (4) always guarantees a perfect fit with *R*^2^ = 1. In addition, the test results that show no displacement rate effect are also excluded from Figure 7.

**Figure 8:**
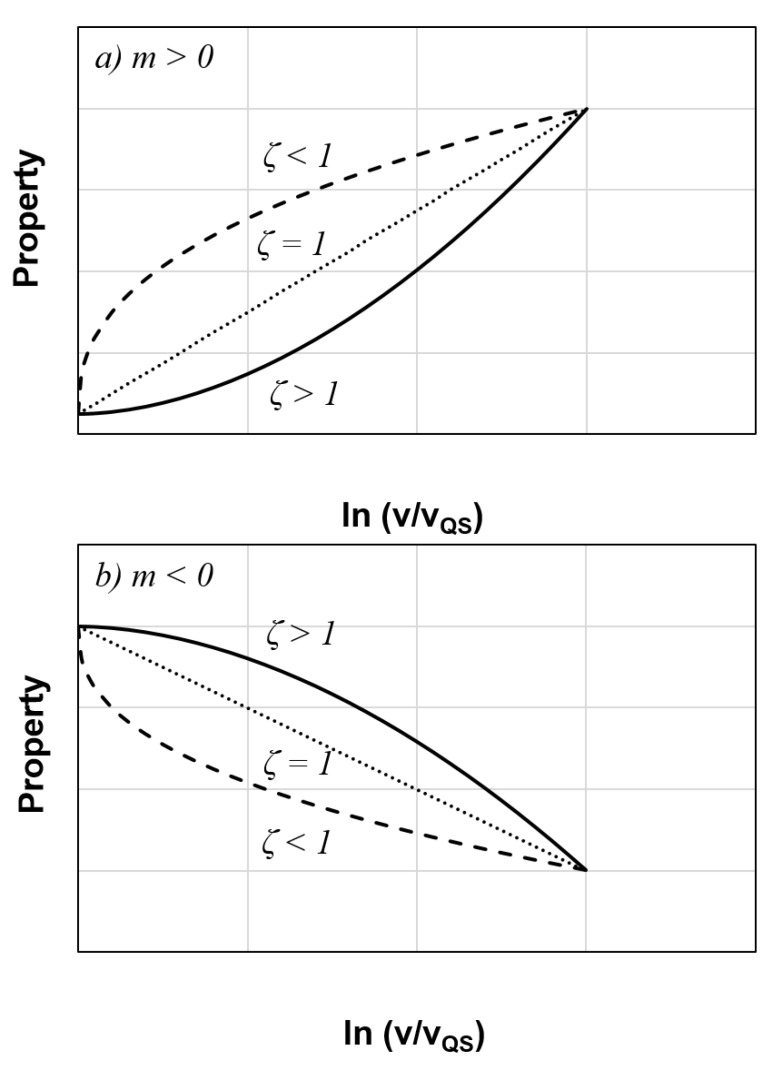
Illustration of the meaning of *m* and *ζ* parameters for (**a**) *m* > 0 and (**b**) *m* < 0.

For clarity purposes, the data are offset vertically from each other in Figure 7. Hence, the first data point of each set of results represents the normalized value of 1. It is worth to note that *m* > 0 indicates that *G_IIC_* rises with displacement rate (Figure 8a), *m* < 0 implies that *G_IIC_* decreases with displacement rate (Figure 8b), and *m* = 0 means that there is no displacement rate effect. On the other hand, the parameter *ζ* describes the trend of variation, where *ζ* > 1 refers to the case having a property which varies exponentially with loading rate, *ζ* < 1 means that the property becomes gradually stabilized at high loading rates and *ζ* = 1 depicts a linear variation. So, Equation (4) is capable of describing most of the mixed test result trends discussed in Section 1.

Table 3 lists the best fit parameters (*m* and *ζ*) and the corresponding *R*^2^ for each data set. It could be seen that most of the results are well fitted, where *R*^2^ > 0.8. For the data sets of “Current study”, “Cantwell (UD C/E)” and “Blackman (AS4 C/PEEK)”, a lower *R*^2^ value is reported due to the non-monotonic trend or scatter in the experimental data. Nevertheless, Equation (4) has generally shown its capability to fit the overall trend on how *G_IIC_* varies with displacement rate. As described in the previous paragraph, the values of *m* and *ζ* provide the information on the variation of the trend. For example, all materials show positive displacement rate effect (*m* > 0) except for “Blackman (AS4 C/PEEK)” (*m* < 0). Furthermore, the values of *m* are relatively small, which indicates the overall variation in *G_IIC_* is not significant. The “Current study” and all three “Cantwell” data sets show a comparatively significant change in *G_IIC_* at low displacement rates (*ζ* < 1). As for “Yasaee” (both “Control” and “Z-pin”), “Colin de Verdiere (Tufted)” and “Blackman (AS4 C/PEEK)” data sets, an exponential change at high displacement rates is noticeable (*ζ* > 1).

## 4. Numerical Modeling

### 4.1. Cohesive Element

**Figure 9:**
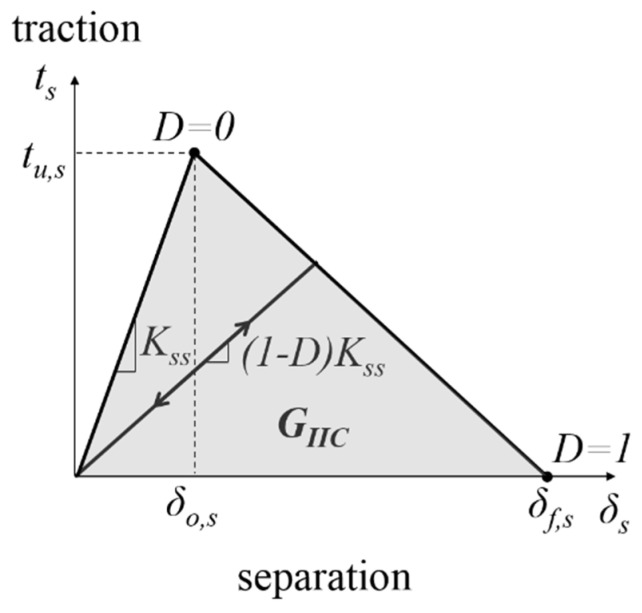
Schematic diagram of the bilinear traction separation law.

Cohesive elements are used to simulate the mode II delamination crack propagation. The mode II traction-separation relationship of the cohesive element is shown in Figure 9. Prior to damage initiation (damage parameter *D* = 0), the uncoupled mode II traction-separation behavior is linear elastic. When the interface shear strength *t_u,s_* is attained, damage is initiated. Upon further loading, damage continues to progress, where 0 < *D* < 1. There is stiffness degradation of the cohesive element as shown in Figure 9. When the total damage energy has reached the interlaminar mode II fracture toughness *G_IIC_*, the cohesive element fails completely and will no longer have load carrying capability (*D* = 1). In this study, linear traction softening is assumed. This is commonly known as the bi-linear traction separation law, which is widely used by various researchers due to its simplicity and accuracy [22,34].

### 4.2. Finite Element Model

**Figure 10:**
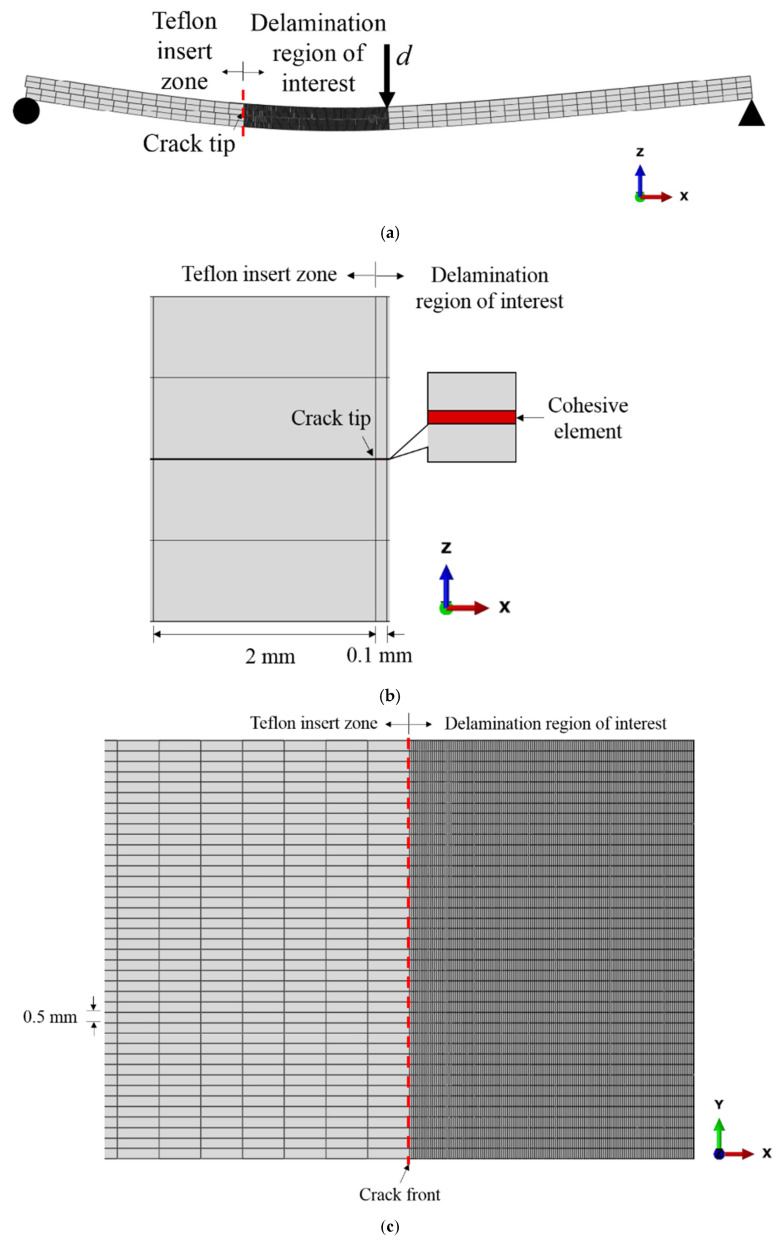
(**a**) Finite element model of the ENF specimen, (**b**) close-up view of the crack tip location (side view) and (**c**) top view near the crack tip location with *y*-axis indicating the width direction.

Figure 10a illustrates the finite element model of an ENF test specimen for mode II delamination. The specimen is simply supported with a vertical displacement applied at the mid span. The loading and boundary conditions as shown in Figure 10a are applied directly on the specimen. Continuum shell elements (SC8R) are used for the composite arms, while cohesive elements (COH3D8) are implemented along the mid-plane interface to simulate delamination propagation. The composite layers are discretized into a total of four elements in the thickness direction which is found to be sufficient to capture the global bending stiffness of the composite [22]. Contact surfaces are defined for the mid-plane surfaces using frictionless contact to prevent interpenetration of the delaminated surfaces. As for the cohesive elements, an element thickness of 10 µm is adopted [35]. The same thickness has been adopted by Mollón et al. [36] and in a previous study [22]. It is also in the same order of the thickness of the Teflon insert employed for the pre-crack and the resin-rich region [37]. The delamination path or delamination region of interest is meshed with fine cohesive element size of 0.1 mm (Figure 10b). From the literature, the typical fracture process zone length for mode II delamination for carbon fiber composites is in the range of 2.5–4.5 mm [38,39]. Using the material properties given in Table 1 and the measured mode II fracture toughness given in Table 4, the cohesive zone lengths estimated using the equations by Harper and Hallett [38] and Soto et al. [40] are within 2.5–3.0 mm and 2.6–3.1 mm, respectively. Therefore, the chosen cohesive element size is sufficient to ensure that the cohesive zone stress distribution within the fracture process zone is correctly and accurately captured. The region outside the delamination region of interest is meshed with a coarser element length of 2 mm (Figure 10b). The width of the specimen (in *y*-direction in Figure 10c is represented by 40 elements with an equal size of 0.5 mm. The simulations are carried out using Abaqus/Standard. 

The displacement rate effect is taken into account by inputting the different mode II fracture toughness values determined experimentally for the four different displacement velocities. In practice, the value of mode II fracture toughness to be used in the numerical model can also be predicted from the proposed Equation (4) for other displacement velocities. This approach is a good approximation for displacement rate of 500 mm/min and below [22].

### 4.3. Cohesive Properties

As shown in Figure 9, the input parameters for the cohesive zone model subjected to mode II shear loading are the shear penalty stiffness *K_ss_*, the interface shear strength *t_u,s_* and the mode II fracture toughness *G_IIC_*. *G_IIC_* is determined from the experiments or can be predicted using the proposed Equation (4). *K_ss_* is estimated using Equation (5).

(5) $$K_{ss}=\frac{E_{m}}{h_{ce}}$$

where *E_m_* = 4.5 GPa (elastic modulus of the epoxy resin [41]) and *h_ce_* = 10 µm (cohesive element thickness). This approach has been successfully implemented in mode II delamination of woven glass/polyester composites [3] and mode I delamination of unidirectional carbon/epoxy composites [22]. It is worth noting that the *K_ss_* = 4.5 × 10^5^ MPa/mm used is also within the range as summarized by Zhao et al. [42] (9.4 × 10^3^–3 × 10^6^ MPa/mm).

The interface shear strength is estimated using Equation (6) [43]:

(6) $$t_{u,s}=\sqrt{\frac{G_{IIC}}{G_{IC}}}t_{u,n}$$

*G_IC_* is the mode I fracture toughness, and *t_u,n_* refers to the interface through-thickness tensile strength. In a separate study on mode I delamination of the same material, it is found that *t_u,n_* = 35 MPa is a good choice for all displacement rates [22]. The value of *t_u,s_* at each displacement rate is therefore assumed dependent on the measured value of *G_IIC_* and will be discussed in Section 4.4.

### 4.4. Numerical Analyses

**Figure 11:**
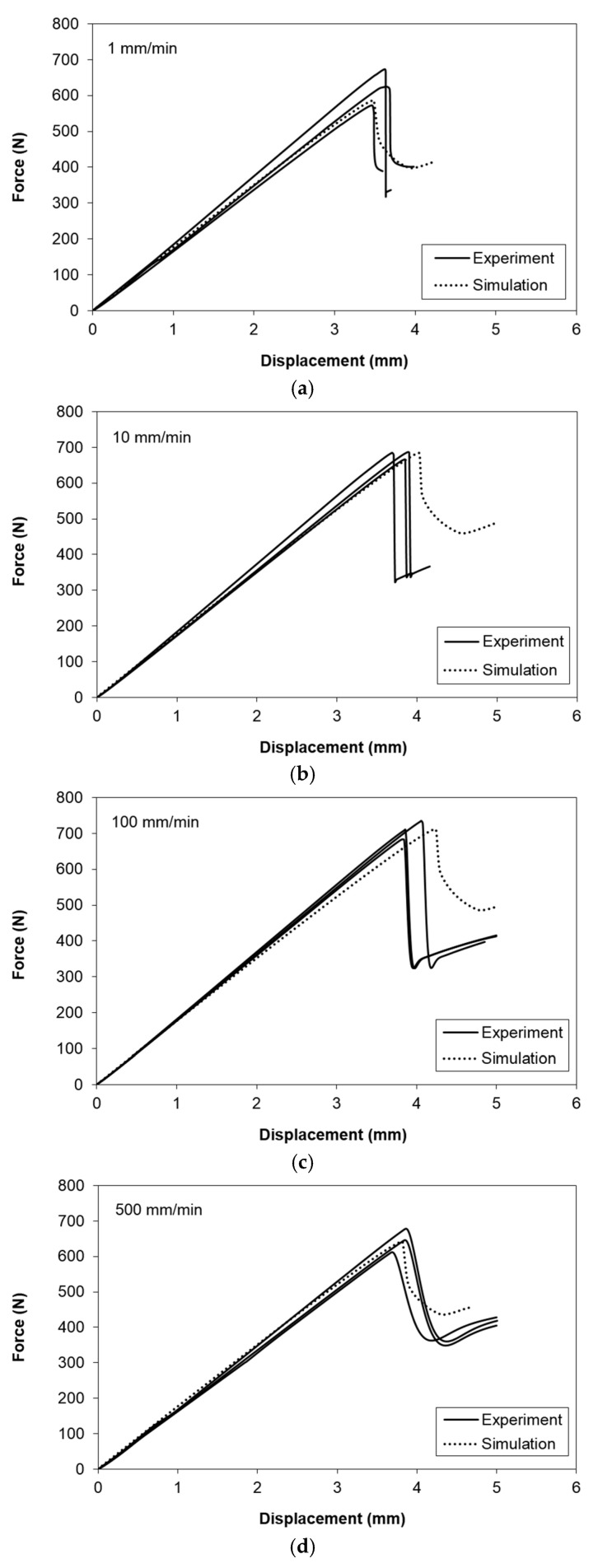
Experimental and numerical force-displacement curves at (**a**) 1 mm/min, (**b**) 10 mm/min, (**c**) 100 mm/min and (**d**) 500 mm/min.

**Figure 12:**
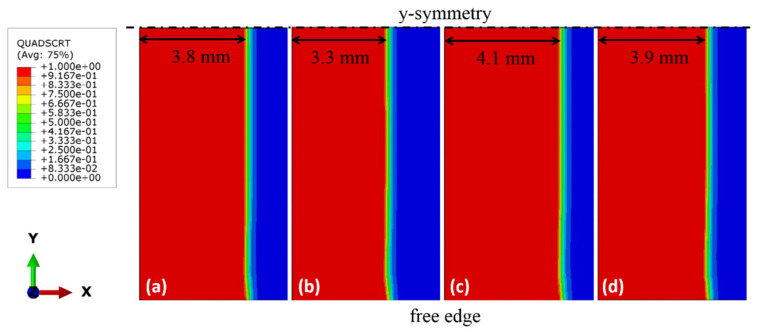
Crack growth profile at peak force for (**a**) 1 mm/min, (**b**) 10 mm/min, (**c**) 100 mm/min and (**d**) 500 mm/min cases.

**Figure 13:**
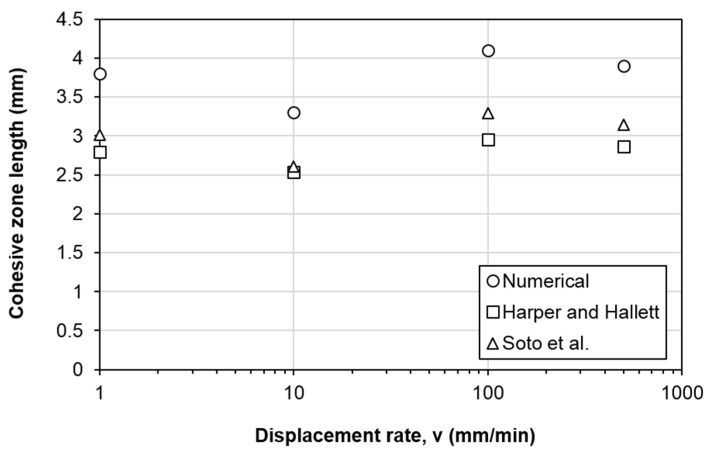
Variation of the cohesive zone length with respect to the displacement rate.

**Figure 14:**
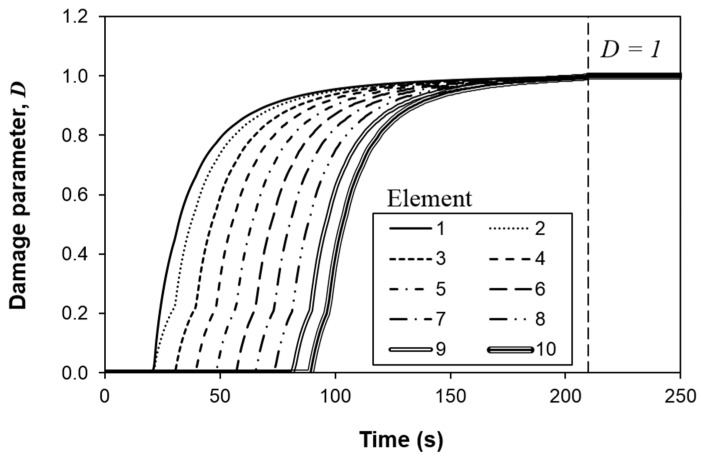
Temporal evolution of the damage parameter of the first ten elements from the initial crack tip.

Figure 11 compares the experimental and numerical force-displacement curves of the ENF specimens at 1, 10, 100 and 500 mm/min, respectively. Simulations are carried out using the interface strengths listed in Table 4 as calculated using Equation (6). Results indicate that both the initial linear slope and the maximum load are in good agreement with the experiments (less than 10%). The largest difference in stiffness is 10% for the 1 mm/min case. As for the maximum load, the difference is 6%, which is also in the 1 mm/min case. This suggests that the methodology adopted in this study is appropriate to simulate the global response of the unidirectional carbon fiber/epoxy composite under mode II loading within the displacement rate of 1–500 mm/min. It is generally recognized that the accuracy of fracture toughness measurement is more important [44], which can be directly obtained from the experiments. This relatively simple methodology provides a guideline to select the appropriate cohesive parameters for accurate simulation, which allows the prediction of crack growth behavior of the specimens that is to be discussed in the following:

Figure 12 depicts the crack growth at different displacement rates when peak force is attained. The display output parameter QUADSCRT reaching 1 (shown in red in Figure 12) indicates that the damage initiation criterion of the cohesive element has been satisfied, i.e. the element is experiencing damage degradation (0 ≤ *D* < 1). So it can be used to indicate the extent or length of cohesive process zone. Due to symmetry, only half of the width is shown. The crack across the width shows a reversed thumb-like pattern, where the crack grows slightly ahead at the specimen edge. This is similar to the observation reported by Koloor et al., [2,20]. At the peak load, damage has been initiated and extended (0 ≤ *D* < 1) from the initial crack tip along the delamination path up to about 3.8 mm, 3.3 mm, 4.1 mm and 3.9 mm at displacement rate of 1 mm/min, 10 mm/min, 100 mm/min and 500 mm/min, respectively, but no element has experienced total damage (*D* = 1) numerically yet. It shows that the cohesive zone length (CZL) of the model is reasonably close to the estimation using the equations by Harper and Hallett [38] (2.8 mm, 2.5 mm, 3.0 mm and 2.9 mm) and Soto et al. [40] (3.0 mm, 2.6 mm, 3.3 mm and 3.1 mm) (Figure 13). It is worth to note that despite the CZLs predicted from the numerical simulation are generally higher than the literature ones, they follow the same trend (Figure 13). The numerical CZLs are closer to the ones predicted by Soto et al. [40]. Figure 14 plots the evolution of damage parameter *D* with respect to time *t* for the first ten elements from the crack tip. It could be seen that other than the first two elements, the damage of the cohesive elements all initiates at a different time despite their subsequent damage evolution is self-similar. However, the complete damage (*D* = 1) for all ten elements happens at the same time (around *t* = 210 s) immediately after the peak load is attained (at *t* = 209 s). The total damage (*D* = 1) of the elements all occurring at the same instant leads to the sudden drop in the sustained load, implying an unstable crack propagation which is common in an ENF test [2,21,45,46,47]. This phenomenon is known as the crack jump [2]. The crack growth behavior at other displacement rates is similar and hence is not further discussed. Analytical models could be considered to analyze interfacial failure in the future [2,20,48,49].

## 5. Conclusions

This research focuses on the experimental and numerical studies of mode II delamination of unidirectional carbon/epoxy composites at displacement rates of 1, 10, 100 and 500 mm/min. The mode II delamination is investigated experimentally using the end-notched flexure (ENF) test. In addition, cohesive zone modeling (CZM) is used to simulate the delamination behavior. Based on the results, it could be concluded that:

1. Mode II fracture toughness *G_IIC_* increases up to 45% at displacement rate 100 mm/min before it drops slightly at 500 mm/min. In general, a higher displacement rate has a positive effect on *G_IIC_* of the unidirectional carbon/epoxy composites used in this study.
2. Surface morphology analyses show that adhesive failure (fiber/matrix debonding) dominates at displacement rate 1 mm/min. At the cases of 10–500 mm/min, similar rough surfaces with matrix hackles are observed, hence the similar magnitude of *G_IIC_* among them. This difference in fracture morphology is postulated to cause the increment in *G_IIC_* at higher displacement rates.
3. The modified rate dependent model (Equation (4)) proposed in this study is found to fit the data from the current study and literature considerably well. Most of the data has *R*^2^ larger than 0.8. So it can be used to describe the mixed effect of loading rate on fracture toughness.
4. The *G_IIC_* value measured from the experiment or given by Equation (4) can be used as the input parameter in the cohesive element model to predict mode II delamination. Numerical analyses indicate that before the peak load is attained, none of the element has been completely damaged. However, once the peak load is reached, total damage is detected concurrently for all elements leading to an abrupt drop in the load and the crack jumps instantaneously. This explains the occurrence of unstable crack propagation.

## Figures and Tables

**Table 1:** Lamina properties for the carbon/epoxy composite used in this study [18].

*E*_11_ (GPa)	*E*_2_ (GPa)	*G*_12_ (GPa)	*G*_13_ (GPa)	*G*_23_ (GPa)	*ν*_12_
103	6.7	2.7	2.7	2.5	0.34

**Table 2:** Mode II interlaminar fracture toughness at various displacement rates, *v*.

*v* (mm/min)	1	10	100	500
*G_IIC_* (N/mm)	1.35 ± 0.17	1.80 ± 0.07	1.96 ± 0.14	1.62 ± 0.17

**Table 3:** Best fit parameters (*m* and *ζ*) and *R*^2^ from the current study and literature.

*Label* (Figure 5)	*m*	*ζ*	*R* ^2^	Reference
Current study	3.29 × 10^−1^	1.36 × 10^−4^	0.7210	/
Yasaee (Control)	1.72 × 10^−3^	2.08	0.8095	[9]
Yasaee (Z-pin)	3.68 × 10^−9^	7.53	0.9570	[9]
Colin de Verdiere (Tufted)	2.14 × 10^−3^	1.88	0.8448	[16]
Cantwell (AS4 C/PEEK)	9.14 × 10^−2^	0.56	0.9061	[11]
Cantwell (PW C/E)	3.27 × 10^−1^	9.10 × 10^−2^	0.8384	[11]
Cantwell (UD C/E)	4.72 × 10^−2^	0.64	0.6883	[11]
Blackman (AS4 C/PEEK)	−5.79 × 10^−9^	6.76	0.5117	[15]

**Table 4:** Interface strength values used for different displacement rate cases.

*v* (mm/min)	*G_IC_* (N/m) ^a^	*G_IIC_* (N/m)	*t_u,n_* (MPa) ^a^	*t_u,s_* (MPa)
1	245.03	1349.26	35	82
10	202.59	1795.78	35	104
100	275.06	1962.69	35	93
500	258.59	1623.85	35	88

^a^ The values of *G_IC_* and *t_u,n_* are taken from the previous study on mode I delamination on the same carbon/epoxy composite at the same displacement rates [18].
